# RE: Someone is to blame: the impact of suicide on the mind of the bereaved (including clinicians)

**DOI:** 10.1192/bjb.2024.84

**Published:** 2024-10

**Authors:** Maria Cecilia Atkins

**Affiliations:** Consultant Psychiatrist, Chair of RCPsych Wales Devolved Council, Royal College of Psychiatrists, UK. Email: maria.atkins@wales.nhs.uk

This article is extremely important and provides an opportunity to begin having rational and psychologically informed conversations about suicide in an increasingly litigious and blaming societal context. The collaboration of individual clinicians with the unhelpful beliefs surrounding suicide has been well described, and the importance of reflective space to recognise such reactions is clear. But there are other examples of collusion in ‘delusional’ beliefs about suicide in the expert opinions used, for instance, in coroners’ inquests. The new Expert Witness Lead in the Royal College of Psychiatrists might have a role in positively influencing the quality of such work. The RCPsych Wales Devolved Council inquest subgroup have been reflecting on the increasingly adversarial nature of coroners’ inquests in recent years. Challenging this as a profession is surely what we must do as a College, in the interests of recruitment and retention if nothing else?

